# Problem gambling, associations with comorbid health conditions, substance use, and behavioural addictions: Opportunities for pathways to treatment

**DOI:** 10.1371/journal.pone.0227644

**Published:** 2020-01-10

**Authors:** Madison Ford, Anders Håkansson

**Affiliations:** 1 Dept of Clinical Sciences Lund, Psychiatry, Faculty of Medicine, Lund University, Lund, Sweden; 2 McGill group for suicide studies, McGill University, Montreal, Québec, Canada; University of Auckland, NEW ZEALAND

## Abstract

**Background:**

Problem gambling is a public health issue and its comorbidity with other health conditions may provide an opportunity for screening in healthcare settings; however, a high level of uncertainty and a lack of research in the field remains. The objective of this study is to investigate potential associations between problem gambling and numerous other health conditions, including substance use, mental health problems, and behavioural addictions.

**Methods:**

A cross-sectional web-survey was distributed by a market research company to an online panel of respondents in Sweden, which aimed to be representative of the general population. Chi-squared and Mann-Whitney U tests, followed by logistic regression analysis, were performed to determine associations between screening positive for lifetime problem gambling and potential comorbid conditions and behaviours.

**Results:**

Among 2038 participants, 5.7 percent screened positive for lifetime problem gambling. Significant associations were found between problem gambling and male gender, education level, daily tobacco use, moderate psychological distress, problematic shopping, and problem gaming.

**Conclusion:**

The association between screening for problem gambling and other health conditions, including psychological distress and behavioural addictions such as shopping and gaming, demonstrates the need to screen for problem gambling in the context of other health hazards, such as in different healthcare settings. Further research is required to identify the temporal relationship between these conditions and to investigate underlying etiological mechanisms.

## Introduction

Problem gambling is a public health concern and is comprised of a spectrum consisting of multiple levels of gambling problems, from at-risk gambling to gambling disorders [1]. The terms ‘problem’ and ‘pathological’ gambling are often used interchangeably, however problem gambling is often used to describe an intermediate or subclinical form of the disorder [2]. The actual clinical diagnosis, recognized as pathological gambling, is defined by the WHO as consisting of “frequent, repeated episodes of gambling that dominate the patient's life to the detriment of social, occupational, material, and family values and commitments” [3]. This diagnosis also corresponds to the term ‘gambling disorder’ which is classified in the Diagnostic and Statistical Manual of Mental Disorders (DSM-5), as “persistent and recurrent problematic gambling behavior leading to clinically significant impairment or distress, as indicated by the individual exhibiting four (or more) of the diagnostic criteria in a 12-month period” [4]. Clinical characteristics of problem gambling include legal problems, reduced quality of life, impaired psychological functioning, and increased rates of bankruptcy, divorce and incarceration [5, 6]. Gambling problems have also been associated with a variety of comorbid disorders that when present have been shown to increase gambling problems and the severity of associated consequences [7, 8].

Previous research has indicated a strong association between problem gambling and various comorbid disorders that include mental health disorders, such as anxiety and mood disorders, substance use and personality disorders, in addition to psychotic spectrum disorders [9, 10]. Despite studies controlling for sociodemographic and socioeconomic characteristics, substantially positive and significant associations between pathological gambling and substance use, mood, anxiety, and personality disorders tend to persist [11]. High prevalence estimates of alcohol use disorder and drug use disorder have also been reported in pathological gamblers, and it is believed that substance use and gambling may share antecedent factors [11, 12]. On the upper end of the range of prevalence estimates of psychiatric disorders in pathological, problem and at-risk gamblers, some studies have reported values greater than 80% [13, 14]. Research on the co-morbidity of physical health problems is much rarer, however have showed that problem gamblers are more likely to avoid regular exercise, have an unhealthy diet, be obese (regarding their body mass index), and experience medical conditions including tachycardia, angina, cirrhosis and other liver disease [15–17]. It is worth noting that these associations persisted once demographic characteristics and behavioral risk factors had been controlled for, including obesity, alcohol abuse and dependence, and nicotine dependence. The poorer health behaviour exhibited by problem gamblers in comparison to non-problem gamblers also includes a higher prevalence of sedentary leisure activity [17]. Problem gamblers are also consistently more likely to rate self-reported aspects of physical health as worse than their non-gambling counterparts [18]. Research patterns have shown that co-morbidity “exacerbates disordered gambling symptomatology and associated functional outcomes”, regardless of the type of comorbid disorder [19].

A lesser researched area of co-morbidity gambling research is the potential link between problem gambling and other non-substance-related addictive behaviours, including internet use, video-game playing, sex, eating, and shopping addictions. Food addiction has been found to have a point prevalence of 9.2% in gambling disorder treatment seeking patients, and has been associated with worse emotional and psychological state than those with a gambling disorder only [20]. A variety of these behavioural addictions are also considered impulse control disorders, including compulsive sexual behaviour and compulsive buying; many of which have been found to be common among pathological gamblers and are associated with more severe gambling symptoms [21], although uncertainty remains with respect to the true prevalence of these conditions [22, 23]. It has previously been established in research on the association of problem gambling and shopping addiction that adolescents with problematic gambling are more likely to suffer from anxiety which could only be relieved by shopping [24]. Problematic gaming has also been found to be positively correlated with problem gambling [25]. Evidence has also been found of an increased prevalence of problem internet use among problem gamblers, as well as those with problem gambling being significantly more likely to report problematic internet consumption [26, 27]. Additionally, internet addiction has been shown to be significantly associated with internet gambling [28, 29]. Similar to the previously discussed comorbidities, results have shown interaction between gambling problems and co-occurring addictive behaviours, more specifically demonstrating a dose-response relationship between the number of co-occurring behaviours and self-reported severity of gambling problems [30].

Though there appears to be a significant amount of research that points to a strong association between problem gambling and various co-morbidities, including substance use and psychiatric disorders [31], there appears to be significant variation between numerous reported prevalence estimates of comorbid conditions. Additionally, the majority of co-morbidity studies have drawn conclusions from treatment seeking samples, which can be unrepresentative of the general problem gambling population, as it has been estimated that only 7–12% of pathological gamblers seek treatment [2, 32]. There may be systematic differences between treatment-seeking and general population gamblers, as evidence suggests that treatment-seeking samples generally display more severe gambling symptoms [32], and consist mostly of pathological gamblers who are white, male and middle-aged [33]. The paucity of research into physical health conditions and non-substance-related behavioural addictions as potential problem gambling comorbidities also elicits reasoning for further research into this area. Thus, the purpose of this study is to investigate the potential association between screening positive for a lifetime history of problem gambling, and various other health conditions, including mental health problems, problematic substance use, physical health conditions, and behavioural addictions. The intent of this research is to determine new opportunities for pathways to effective treatment through the integration of problem gambling screening in other contexts where related health problems are treated.

## Methods

### Study sample

Survey participants were recruited for the study through the company Userneeds, which distributed the quantitative web-survey to participants in a pre-existing online web panel of survey respondents. Userneeds web panel [34] consists of a total of 300,000 panel participants across the six countries they operate in, including Denmark, Norway, Sweden, Finland, Ireland, and Switzerland. The current study, however, solely addressed participants living in Sweden. Individuals were recruited to achieve accurate representation of the distribution of sex and age in the general Swedish population, aiming to cease data collection once approximately 2,000 respondents had been reached. By their own volition, the web panel participants had previously signed up and agreed to receiving invitations to regular surveys via e-mail by providing the Userneeds system with their personal information. As consenting members of the web panel, individuals were invited to respond to the present survey and chose to participate or not, therefore making participation in the study completely voluntary. The Swedish Userneeds web panel specifically consists of 115,000 individuals above 16 years of age, currently living in Sweden, who through Userneeds have agreed to receiving questionnaires for different types of inquiries. In the type of data collection used here, it cannot be established how many individuals were contacted in order to achieve the targeted number of participants, but 17 individuals were recorded to have read the information without entering the survey, and a total of 123 individuals proceeded to the survey without completing it.

Data collection for the current study was carried out for the duration of five days in April, 2019. Participants below the legal gambling age of 18 were excluded from recruitment into the research study. Of the 2170 participants in the survey, only those who completed all questions related to the inclusionary criteria for at risk/problem gambling, and completed all questions related to substance use disorders, mental health disorders and behavioural addictions were included. From this total of responses, total of 2045 complete responses were received; however, seven of these potentially could have been duplicate answers originating from the same IP address and from the same gender and age group, and the second one of these responses was therefore excluded from further analysis. Thus, a final sample of 2038 participants were included in the study.

### Procedures

Prior to distribution, the survey was first drafted in English by the researchers and then officially translated into Swedish to comply with the official language of the country. Furthermore, once its contents were complete the survey was then designed for online use by two collaborating companies, Patent Information Broker Ltd (PIB) and I-Mind Consulting Ltd (I-mind). Survey invitations were distributed to members of the Userneeds web panel containing information regarding the study subject matter and participation in the study. Each panel member was informed in writing that the present study addressed gambling, addiction to gambling and mental, behavioral and substance use disorders. The anonymity of the study was also explicitly stated, in addition to confirming that answers would be handled with strict confidentiality. Members of the web panel received credits in the company’s bonus system, corresponding to a value of approximately one euro upon completion of one survey in the system. Following the processing of the information provided in the email, when participants opened the link to the survey, they were required to electronically provide an informed consent, only after which the survey would open for completion.

Anonymity of participants was ensured through a process involving the employment of the company PIB and the careful determination of included variables. Userneeds, who were responsible for the distribution of the questionnaires, were aware of the identity of the individuals who are part of their web panel, therefore PIB was employed to collect the data from the questionnaires, as they were unable to obtain the identity of any participant. Once the data was collected by PIB it was analyzed by the authors. Variables included in the survey were confirmed to not reveal the identity of study participants directly or indirectly by grouping information about age in intervals, and avoiding the collection of any personal information, information about geographical area or other potentially identifiable data.

Ethics approval was obtained from the Regional Ethical Review Board in Lund, Sweden (file number 2019–01830).

### Measures

#### Sociodemographic data

Variables pertaining to this category included in the survey were age, gender, primary occupation, education, income, marital status, and place of birth. The variables chosen were based on the relevant variables considered in previously conducted studies [19]. Age was stratified in a total of six brackets which consisted of 18–24 years old, 25–29, 30–39, 40–49, 50–59, and 60 or older. Gender was categorized as woman, man, and transgender although a ‘prefer not to answer’ choice was also available for the potentially sensitive nature of the questions. Primary occupation options consisted of studying, working, job seeking, retired, house wife/husband, sick leave, other. Highest level of education achieved included the categories elementary school, high school, university studies without full qualification, completed university degree, other. Monthly income was in Swedish currency (SEK) and divided into ten brackets consisting of less than 10,000, 10,000–15,000, 15,000–20,000, 20,000–25,000, 25,000–30,000, 30,000–35,000, 35,000–40,000, 40,000–45,000, 45,000–50,000, and higher than 50,000 (one SEK corresponding to around 0.09 Euros). Marital status was categorized as single, married/partner/partnership, widow/widower, and divorced/separated. Place of birth was categorized as Sweden, other Nordic country (Denmark, Norway, Finland, Iceland), other country in Europe, other country outside of Europe.

#### Physical health

The physical health status of participants was measured with questions regarding physical health in general, physical activity, and BMI. General physical health was evaluated with the question “how would you describe your physical health in general?”, with answers in the form of a Likert scale ranging from excellent to very poor. To assess participants level of physical activity the following question was included “in a typical week, how many days are you involved in any heart-pounding activity in order to improve your fitness and your well-being?”, with numeric answers that ranged from no days to 7 days a week (everyday). To provide clarity and ensure the validity of the answer, a variety of potential activities were also listed as part of the question. Evidence on single question physical activity assessment has shown it is an acceptable alternative to more detailed measures in studies that are not primarily focused on physical activity [35]. BMI was calculated for all participants by dividing their approximate self-reported weight by their height squared, measured in kilograms and meters, respectively, and treated as a continuous variable. As height and weight were reported in categories rather than with the exact figures (due to the format of an online survey), the mid-value of each category was chosen for analysis.

#### Substance use

The Alcohol Use Disorders Identification Test (AUDIT-C) was used to assess participants’ past-year alcohol consumption by identifying hazardous drinkers and/or those that have active alcohol use disorders, including alcohol abuse or dependence [36]. The brief 3-item screening tool is a modified version of the 10-item AUDIT instrument that asks questions about alcohol consumption levels and patterns in the past 12 months, and the summed score ranges from 0 to 12. The AUDIT-C has been reported to have a high validity in Swedish populations, as it had an excellent ability in detecting dependence and risk drinking [37]. The tool has a sensitivity of 0.78 and specificity of 0.69 in men and a sensitivity of 0.80 and a specificity of 0.72 in women for identifying any alcohol use disorder or risk drinking [37]. To measure the degree of hazardous health behaviours on a sub-diagnostic level, total scores were treated as continuous variables, rather than using cut-off points.

Drug use (with a past-year time frame) was addressed by the question “in the past year have you used an illegal drug or a prescription medication for non-medical reasons?”, also specifying a list of specific drugs that would meet these criteria to ensure validity. Evidence has shown the accurate identification of drug use and drug use disorder by the single screening question, which has been found to possess similar test characteristics to those of longer screening tools [38]. Furthermore, the question has a sensitivity of 100% and a specificity of 73.5% for the detection of a drug use disorder [38]. An additional question regarding nicotine dependence asked if the participant smoked or took snuff daily, to which they had the option to reply ‘yes’ or ‘no’, in order to assess a likely and current tobacco use disorder.

#### Mental health

As a broad screener for psychological distress, the Kessler Psychological Distress Scale (K6), a 6-item self-reported tool, was used with the intention of assessing risk for serious mental illness in the general population and is based on questions about anxiety and depressive symptomology. Each question addresses how often the respondent had six different feelings or experiences in the past 30 days, which include questions about being “nervous,” “hopeless,”, “restless or fidgety,” “so depressed that nothing could cheer you up,” “that everything was an effort,” and “worthless”. The total score ranges from 0 to 24, based on each item being coded 4 (all of the time), 3 (most of the time), 2 (some of the time), 1 (a little of the time), or 0 (none of the time) [39]. The optimal cut-off points have been determined to be a K6 score of ≥13 as the upper threshold, indicative of severe mental illness, and K6 ≥5 as the lower threshold, indicative of moderate mental distress [40]. The severe psychological distress score of ≥13 has a sensitivity of 0.36 and a specificity of 0.96, with a total classification accuracy of 0.92, while the moderate psychological distress score of ≥5 has a sensitivity of 0.76 and specificity of 0.75 [39, 40]. In the present study, 24 individuals had missing data for one or two K6 items. In the addition of the summed score for these individuals, the median values of the available items were imputed for the missing items.

In addition, mental health was also evaluated with a question about whether a doctor had ever prescribed them prescribed medication or therapy as a treatment for mental illness, to which they could reply ‘yes’ or ‘no’.

#### Problem gambling

A modified version of the NORC DSM-IV Screen for Gambling Problems (NODS), referred to as the NODS-CLiP, was used to screen for a lifetime history of problem or pathological gambling. The “CLiP” refers to the contents of the survey which includes three NODS questions pertaining to loss of Control, Lying, and Preoccupation, including “have there ever been periods lasting two weeks or longer when you spent a lot of time thinking about your gambling experiences or planning out future gambling ventures or bets?”, “have you ever tried to stop, cut down, or control your gambling?”, and “have you ever lied to family members, friends, or others about how much you gamble or how much money you lost on gambling?”. Answering ‘yes’ to one or more questions indicates the presence of problem or pathological gambling at some time during the individual’s lifetime. The NODS-CLiP has been found to be highly efficient and effective identifying problem gamblers, evidenced by a sensitivity of 0.94–0.99 and specificity 0.88–0.95 [41].

#### Behavioral addictions

Problematic internet use was assessed using the 3-item Problematic and Risky Internet Use Screening Scale (PRIUSS-3) [42]. The questions are asked in present tense, and include the following; “how often do you experience increased social anxiety due to your Internet use?”, “how often do you feel withdrawal when away from the Internet?”, and “how often do you lose motivation to do other things that need to get done because of the Internet?”, to which the response options response options utilize a Likert scale with scores of 0 through 4 (0 = never; 1 = rarely; 2 = sometimes; 3 = often; 4 = very often). The PRIUSS-3 has been shown to possess a sensitivity of 0.98 and a specificity of 0.80 [42]; proving its validity as a screening instrument.

The 7-item Bergen Shopping Addiction Scale (BSAS) was the tool selected to assess shopping addiction through the use of core addiction criteria, including salience, mood modification, tolerance, withdrawal, conflict, relapse, and resulting problems. Each of the seven questions reflect one of the seven aforementioned elements of addiction, “thus ensuring its content validity in an addiction framework” [43]. The questions were in the form of statements, expressed in present tense, to which respondents could choose ‘completely disagree’ (0), ‘disagree’ (1), ‘neither disagree nor agree’ (2), ‘agree’ (3), and ‘completely agree’ (4) [43]. The seven statements were “I think about shopping/buying things all the time”, “I shop/buy things in order to change my mood”, “I shop/buy so much that it negatively affects my daily obligations (e.g., school and work)”, “I feel I have to shop/buy more and more to obtain the same satisfaction as before”, “I have decided to shop/buy less, but have not been able to do so”, “I feel bad if I for some reason are prevented from shopping/buying things”, and “I shop/buy so much that it has impaired my well-being”. Previous research has concluded that the BSAS “has good psychometrics, structure, content, convergent validity, and discriminative validity” [43].

To assess the presence of gaming addiction in the sample, the Gaming Addiction Scale (GAS) was used. Similar to the BSAS, the scale uses the seven DSM-based criteria for addiction, with each of the seven items pertaining to one criterion, and response options being in the form of a Likert scale ranging from ‘never’ to ‘very often’ (1–5 points). This scale assesses the last six months, and includes “did you think about playing a game all day long?”, “did you spend increasing amounts of time on games?”, “did you play games to forget about real life?”, “have others unsuccessfully tried to reduce your game use?”, “have you felt bad when you were unable to play?”, “did you have fights with others (e.g., family, friends) over your time spent on games?”, and “have you neglected other important activities (e.g., school, work, sports) to play games?”. In previous research the GAS showed high reliabilities and good concurrent validity across various samples [44].

### Statistical analysis

All statistical analyses were conducted with the utilization of IBM SPSS Statistic version 25.0. A bivariate analysis was first performed in order to compare the prevalence of the sociodemographic variables and co-occurring conditions of interest amongst those who did or did not screen positive for a history of problem gambling (using chi-squared tests for categorical variables and the Mann-Whitney U test for the AUDIT-C, PRIUSS-3, BSAS, and GAS, as well as BMI AUDIT-C, PRIUSS-3, BSAS, and GAS). As the K6 screening tool has the ability to identify both moderate and severe levels of psychological distress, this was analyzed as a categorical variable, rather than utilizing the total scores as a continuous variable. Although the study included participants with complete survey data, certain variables, those regarding gender, tobacco use, drug use, a history of being prescribed treatment for psychological distress, provided a ‘prefer not to answer’ option. Thus, individuals with a missing answer for any of these variables were excluded from the logistic regression analyses, which therefore included a total of 2,012 and 2,009 individuals, respectively. To examine possible associations between problem gambling and the comorbidities of interest, as well as adjust for potential confounders, binary logistic regression analyses were performed, where odds ratios (OR) with 95% confidence intervals (CI) were calculated. Prior to running a binary logistic regression analysis, a correlation matrix was created and examined to test for the potential presence of multicollinearity between the independent variables to be entered in the logistic regression. Statistical relationships were identified as being statistically significant by producing a p value <0.05.

## Results

A total of 5.7% (n = 116) screened positive for a lifetime history of problem gambling, and its associations with separate sociodemographic traits are presented in Table 1. In the sample population, 54.7% identified as female, 45.0% as male, and 0.1% as transgender. The age distribution was considerably representative of the Swedish population with 11.9% being 18–29, 56.3% being 30–59 and 31.8% being above 60. A significant association was found between a positive screen for a lifetime history of problem gambling and younger age (p = 0.002), male gender (p<0.001), lower level of education (p = 0.010) and single marital status (p = 0.018).

**Table 1:** Total frequencies of sociodemographic traits in sample population, prevalence of at-risk/problem gambling for various variable groups, and statistical associations between variable groups and at-risk/problem gambling calculated with the chi-squared test. Data was collected through a survey conducted in Sweden with a total sample size of 2045 participants.

	*Variables*	*No problem gambling (n = 1922)*, *n(%)*	*Problem gambling (n = 116)*, *n(%)*	*p-value*
Age	18–24	73 (4)	6 (5)	0.002*
	25–29	150 (8)	13 (11)	
	30–39	339 (18)	25 (22)	
	40–49	426 (22)	34 (29)	
	50–59	308 (16)	15 (13)	
	60+	626 (33)	23 (20)	
Gender ^a^	Woman	1073 (56)	41 (35)	<0.001*
	Man	843 (44)	75 (65)	
	Transgender	3 (0)	0	
	Rather not answer	3 (0)	0	
Primary Occupation ^b^	Studying	114 (6)	8 (7)	0.479
	Working	1212 (63)	82 (71)	
	Job seeking	49 (3)	2 (2)	
	Retired	456 (24)	17 (15)	
	House wife/husband	17 (1)	1 (1)	
	Sick leave	54 (3)	6 (5)	
	Other	20 (1)	0	
Highest level of education achieved ^c^	Elementary school	102 (5)	7 (6)	0.010*
	High school	691 (36)	52 (45)	
	Incomplete university degree	291 (15)	19 (16)	
	Completed university degree	756 (39)	30 (26)	
	Other	82 (4)	8 (7)	
Monthly Income (SEK)	Less than 10,000	137 (7)	9 (8)	0.772
	10,000–15,000	185 (10)	16 (14)	
	15,000–20,000	178 (9)	8 (7)	
	20,000–25,000	208 (11)	10 (9)	
	25,000–30,000	297 (15)	21 (18)	
	30,000–35,000	311 (16)	21 (18)	
	35,000–40,000	210 (11)	14 (12)	
	40,000–45,000	135 (7)	5 (4)	
	45,000–50,000	92 (5)	4 (3)	
	Higher than 50,000	169 (9)	8 (7)	
Marital status ^d^	Single	448 (23)	41 (35)	0.018*
	Married/partner/partnership	1328 (69)	68 (59)	
	Widow/widower	28 (1)	0	
	Divorced/separated	118 (6)	7 (6)	
Place of Birth ^e^	Sweden	1767 (92)	105 (91)	0.070
	Other Nordic country (Denmark, Norway, Finland, Iceland)	50 (3)	0	
	Other country in Europe	65 (3)	5 (4)	
	Other country outside of Europe	40 (2)	6 (5)	

* Indicative of significant associations.

^a^ Due to nominal characteristic of variable, chi-square test was conducted with males vs. women and transgender individuals

^b^ Due to nominal characteristic of variable, chi-square test was conducted with job seeking and sick leave vs. all other options (including studying, working, retied, house wife/husband, and other).

^c^ Due to the uncertainty of ‘other’ category, chi-square test was conducted with any kind of university education (incomplete and complete) vs. all other options (including elementary school, high school, and other).

^d^ Due to nominal characteristic of variable, chi-square test was conducted with married/partner/partnership vs. all other options (including single, widow/widower, and divorced/separated).

^e^ Due to nominal characteristic of variable, chi-square test was conducted with Sweden and other Nordic countries vs. all other options (including other country in Europe and other country outside of Europe).

In Table 2, various co-occurring health conditions are compared between lifetime problem gamblers and others. Several of these health conditions were significantly associated with screening for problem gambling; past-year drug use (p<0.001), daily tobacco use (p<0.001), lower degree of psychological distress (p<0.001), a history of having felt the need to seek treatment for mental health (p = 0.006), and a history of being prescribed treatment for mental illness (p = 0.002, Table 2). A significant difference was found between the total scores of those with and without at-risk/problem gambling on the AUDIT-C (p<0.001), PRIUSS-3 (p<0.001), BSAS (p<0.001), and GAS (p<0.001) screening tools (Table 3).

**Table 2:** Total frequencies of co-occurring health conditions in sample population, prevalence of at-risk problem gambling for various comorbid groups, and statistical associations between categorical health conditions and at-risk/problem gambling calculated with the chi-squared test (linear-by-linear for variables with more than one option). The statistical association for the continuous BMI variable and at-risk/problem gambling was calculated with the Mann-Whitney U test. Data was collected through a survey conducted in Sweden with a total sample size of 2038 participants.

	*Variables*	*No problem gambling (n = 1922)*, *n(%)*	*Problem gambling (n = 116)*, *n(%)*	*p-value*
How would you describe your physical health in general?^a^	Very poor	18 (1)	2 (2)	0.460
	Poor	198 (10)	18 (16)	
	Good	893 (46)	52 (45)	
	Very good	637 (33)	28 (24)	
	Excellent	176 (9)	16 (14)	
In a typical week, how many days are you involved in any heart-pounding activity in order to improve your fitness and your well-being?	None	278 (14)	16 (14)	0.473
	1 day	269 (14)	21 (18)	
	2 days	339 (18)	24 (21)	
	3 days	382 (20)	20 (17)	
	4 days	237 (12)	9 (8)	
	5 days	193 (10)	14 (12)	
	6 days	88 (5)	4 (3)	
	7 days (everyday)	136 (7)	8 (7)	
BMI score (median)		26.03 (22.98–29.37)	26.29 (24.34–30.79)	0.110
In the past year, have you used an illegal drug or a prescription medication for non-medical reasons?	Yes	46 (2)	10 (9)	<0.001*
	No	1873 (97)	105 (91)	
	Rather not answer ^b^	3 (0)	1 (1)	
Do you smoke or take snuff daily?	Yes	312 (16)	36 (31)	<0.001*
	No	1606 (84)	79 (68)	
	Rather not answer^b^	4 (0)	1 (1)	
Has a doctor had ever prescribed you prescribed medication or therapy as a treatment for mental illness?	Yes	501 (26)	45 (39)	0.002*
	No	1406 (73)	69 (59)	
	Rather not answer^b^	15 (1)	2 (2)	
Kessler Psychological Distress Scale (K6)	No psychological distress	1174 (61)	37 (32)	<0.001*
	Moderate psychological distress	618 (32)	56 (48)	
	Severe psychological distress	130 (7)	23 (20)	

* Indicative of significant associations.

^a^ Due to low numbers reporting very poor health, p values were calculated assessing ‘poor’ and ‘very poor’ health together.

^b^ p value calculated for affirmative answer vs all others

**Table 3:** Associations between at-risk/problem gambling and various comorbid conditions, including hazardous alcohol use (AUDIT-C), problematic internet use (PRIUSS-3), problematic shopping (BSAS), and problem gaming (GAS). Comparisons are made using the Mann-Whitney U test.

*Screening tool*	*Non-problem gamblers (n = 1922)*	*Problem gamblers (n = 116)*	*P-value*
AUDIT-C	3 (1–4)	4 (2–6)	*<0.001
PRIUSS-3	1 (0–3)	3 (1–5)	*<0.001
BSAS	1 (0–4)	6 (1–13)	*<0.001
GAS	7 (7–8)	10 (7–15)	*<0.001

* Indicative of significant associations.

In the first logistic regression model that included the comorbidity variables, screening for lifetime problem gambling appears to be significantly associated with daily tobacco use, hazardous alcohol use, problematic shopping, and problem gaming. In the second model that included the comorbid variables and adjusted for the significant sociodemographic variables, lifetime problem gambling remained associated with daily tobacco use, problematic shopping, problem gaming, moderate psychological distress, male gender, and lower level of education (Table 4).

**Table 4:** Associations between at-risk/problem gambling and various comorbid condition variables, including drug use, daily tobacco use, psychological distress, history of being prescribed treatment for mental illness, hazardous alcohol use (AUDIT-C), problematic internet use (PRIUSS-3), problematic shopping (BSAS), and problem gaming (GAS), as well as sociodemographic traits, including age, gender, education, and marital status. Statistical associations were calculated with binary logistic regression analysis.

*Health condition*	*Variables*	*Model 1 OR (95% C*.*I*.*)*, *n = 2*,012**	*Model 2 OR (95% C*.*I*.*)*, *n = 2*,009***
Drug use		1.107 (0.442–2.776)	1.131 (0.447–2.861)
Daily tobacco use		*1.960 (1.209–3.179)	*1.707 (1.047–2.783)
Psychological distress (K6)	None	(ref).	(ref).
	Moderate	1.544 (0.931–2.562)	*1.796 (1.064–3.031)
	Severe	1.438 (0.676–3.062)	1.709 (0.773–3.776)
Ever prescribed medication or therapy as treatment for poor mental health?		0.918 (0.576–1.461)	1.159 (0.705–1.905)
Hazardous alcohol use (AUDIT-C score)		*1.111 (1.015–1.216)	1.078 (0.986–1.179)
Problematic internet use (PRIUSS-3 score)		1.069 (0.959–1.193)	1.117 (0.998–1.249)
Problematic shopping (BSAS score)		*1.076 (1.038–1.116)	*1.088 (1.048–1.130)
Problem gaming (GAS score)		*1.146 (1.088–1.207)	*1.131 (1.072–1.194)
Age	18–24	-	0.612 (0.197–1.902)
	25–29	-	0.577 (0.227–1.465)
	30–39	-	0.716 (0.344–1.489)
	40–49	-	0.970 (0.528–1.782)
	50–59	-	0.918 (0.451–1.872)
	60+	-	(ref).
Male gender		-	
		-	*2.849 (1.730–4.691)
University education		-	
		-	*0.587 (0.380–0.909)
Marital status (married/partner/partnership)		-	
		-	0.745 (0.481–1.155)

* Indicative of significant associations.

**Nagelkerke 0.204

***Nagelkerke 0.251

## Discussion

The aim of the present study was to determine the potential association between a positive screen for a lifetime history of problem gambling and various co-occurring health conditions, specifically investigating, substance use, mental health problems, and behavioural addictions. The results demonstrated a significant association between problem gambling and numerous variables of interest, including daily tobacco use, moderate psychological distress, problematic shopping, problem gaming, when controlling for one another, as well as other potential risk factors for problem gambling, such as age. Also, problem gambling was associated with male gender and with a lower level of education. The findings of the present study demonstrate the importance of integrating screening and treatment of problem gambling into prevention programs and healthcare interventions in settings where related health problems are addressed. Somewhat surprisingly, no association was seen between a positive problem gambling screen and the measures of overweight, physical exercise, or overall physical health.

### Sociodemographic variables

In the final logistic regression model, those who identify as male and have less than a university level education had significantly greater odds of screening positive for a history of problem gambling. These findings are in accordance with results from a recent French general population survey that found the typical problem gambler in this population was “male, aged predominantly between 25 and 64 years old, single, unemployed, and perceive their financial situation as difficult” [45], and it is well established in the literature that male gender is a risk factor for harmful gambling, as well as less formal education [46, 47]. This could be explained by the results of a study examining the gender difference for gambling problems, which found that men had lower levels of impulsive coping, were more socially anxious, and took more risks than women, and that those who had a pronounced demonstration of these traits were likely to engage in gambling [48].

### Physical health

In the present study, poor self-reported physical health, including obesity classification, and health-related lifestyle choices regarding physical activity were not associated with a lifetime history of problem gambling. The lack of association found between a history of problem gambling and current obesity is surprising, as multiple studies using population-based surveys have shown associations between these conditions [15–17, 45]. For example, a more sedentary lifestyle in problem gamblers has been described (17), whereas in contrast, in the present study, no significant association could be seen with the frequency of exercise, with the BMI, or with general subjective physical health. The contrast between the present study and previous research may be due to the smaller sample size used in comparison to those of other general population studies, therefore the power of the significance tests may not have been strong enough to demonstrate such relationships. Additionally, a more elaborate assessment tool may have been required to identify such associations, as few questions were included and BMI could not be calculated precisely, due to the need for technical reasons in the online survey to identify absolute categories and not exact measures of height and weight, making conclusions difficult to be drawn. More research is needed which highlights the potential associations not only between gambling and psychological and behavioural measures, but also with physical health hazards.

### Substance use

Daily tobacco use was statistically associated with problem gambling, and problematic past-year alcohol consumption, measured with the AUDIT, was significantly more common in the problem gambling group, and although this association remained when controlling for other addictive behaviours, it did not remain significant when controlling also for socio-demographic variables. Likewise, past-year drug use was markedly more common in the problem gambling group, although this association disappeared when controlling for other variables in the regression models. The lack of an independent association with drug use is in contrast to previous studies that have found evidence of this association [9]; one study demonstrating individuals with a drug use disorder to be 3.5 times more likely to be a problem gambler [11]. The lack of significance could be explained by the limited number of questions included in the survey regarding drug use, as a single question was used which may have been insufficient to capture the association.

The findings of a link between current tobacco smoking and problem gambling corroborate prior research regarding alcohol and tobacco use disorders in problem gambling, reporting high prevalence estimates of 60%, and with a prevalence of alcohol use disorders reported in as many as 73% [11, 12]. In terms of temporality, it has been reported that issues with substance use, including alcohol problems, tobacco use and cannabis use, commonly began previous to the development of a gambling problem [49]. There may be several explanations for substance use preceding a gambling problem, including the relation to the underlying mechanism of impulse control issues resulting in increased risk taking with respect to both gambling and substance use [17]. It has been proposed that the development and maintenance of gambling and problematic substance use may be related in terms of individuals with these conditions possessing shared predispositions, including those involving genetic, environmental, and social factors, as well as similar personality profiles [2, 50]. Multiple twin studies have demonstrated shared genetic and environmental risk factors for disordered gambling and alcohol use disorders, resulting in a common vulnerability of experiencing these conditions [51, 52]. Additionally, there is a resemblance between the neurocognitive functioning in pathological gambling and alcohol dependence, indicating a possible common neurocognitive etiology [53]. Individuals with a substance use disorder may also feel obliged to continue their gambling behaviour to finance the sustained consumption of the addictive substance [17]. Conversely, if the gambling problem precedes the substance use, the individual may be utilizing the substance as a way of coping with the psychological distress caused by gambling problems and as an escape from their gambling losses [17, 45].

In the present study, using screening tools in an online survey where the extent of the questionnaires was limited, potential temporality between substance use and gambling should be discussed with caution; the screening measure for problem gambling described a lifetime situation, whereas the wording of the tobacco question addressed whether the individual was currently a daily smoker, and likewise, for the alcohol and drug use measures, these refer to the past year. Thus, this fact limits the conclusions to be drawn about exactly how these conditions may be related to one another, but again, in accordance with previous literature, indicate the association between tobacco smoking and problem gambling even when controlling for a number of socio-demographic factors and other types of addictive behaviours.

### Mental health

The K6 diagnostic tool, in the non-adjusted analyses, indicated that problem gamblers were considerably more likely to screen positive for recent (past-month) moderate or severe psychological distress scores indicative of moderate mental distress in comparison to non-problem gamblers. Even when controlling for other variables in the logistic regression, the association with moderate psychological distress (although not with severe psychological distress), persisted. This finding confirms results from previous studies that showed psychological distress to highly increase the risk of screening positive for problem gambling [45, 54, 44], and studies demonstrating high rates of psychiatric comorbidity in problem gambling [2, 9–11]. Research on the temporality of problem gambling and mood disorders have found that 70% of participants reported their disorder to precede the development of their gambling problems, and a proposed explanation for the association is that maladaptive coping strategies are employed by using excessive gambling as a method of escape from symptoms [55, 56, 57]. These findings tie into the well-established ‘pathways model’ of problem gambling in terms of the ‘emotionally vulnerable problem gambler’, as this predictive sub-type is proposed to be a manifestation of poor coping and problem-solving skills that ultimately uses gambling as an emotional escape from premorbid disorders [58, 59]. The use of gambling to make symptoms of psychological problems milder by serving as a function of escape is referred to as ‘self-medication’ and is a factor that could explain gambling problems succeeding mental health related comorbidities [60, 61]. Conversely, gambling has also been hypothesized to lead to depression, due to the negative consequences disordered gambling can have on family and financial issues [62, 63]. Likewise, the pathways model also includes the explanatory model of an ‘antisocial impulsivist gambler’, which has been bolstered by evidence of a significant correlation between psychological distress and impulsivity, as well as between impulsivity and pathological gambling [58]. Severe psychological distress, indicating severe mental illness, was not found to have a statistically significant association with a history of problem gambling in the final logistic regression model when adjusting for the other comorbidities and sociodemographic factors. Though strong associations between problem gambling and psychiatric disorders have been demonstrated in clinical settings [64], one interpretation of the lacking association in the present study may be the low absolute number of respondents reporting both problem gambling and a high level of psychological distress. In the category combining problem gambling and severe mental illness in the general population sample.

Although associations did not hold for an independent association with the higher degree of psychological distress, it remains of great relevance to address problem gambling in patients assessed for mental health disorders. Additionally, it remains crucial to address symptoms of mental health in the assessment of patients seeking help for problem gambling. Importantly, the risk of reporting psychological distress on a severe level was almost three times higher in those screening positive for a lifetime history of problem gambling. Thus, this continues to support the need to address gambling and mental health in the same or strongly coordinated treatment settings and to implement structured screening programs with respect to each of these conditions.

### Behavioural addictions

All three behavioural addictions assessed here were found to display clearly higher scores in individuals screening positive for problem gambling. The associations remained for current problematic shopping and past-six-month gaming, even after adjusting for gender, age, marital status and education. This is consistent with previous research that identified a considerable prevalence of shopping addition in pathological gamblers, estimated by one study at 8% [22], as well as a positive correlation between problem gaming and problem gambling [25]. Likewise, consistent with previous research, current problematic internet use was found to be significantly more common in lifetime problem gamblers [26, 27], although in the final logistic regression model, this association merely lost its statistical significance. The validity of ‘problematic internet use’ as a disorder is controversial, as researchers believe individuals may be using the medium of the internet to engage in specific addictive behavior, such as online gaming and shopping, stating that “internet addicts are no more addicted to the internet than alcoholics are addicted to bottles” [65]. Also, the present associations should be seen in light of the increasing share of gambling happening online; thus, this may further explain the link between screening positive for a problematic internet behaviour and problematic gambling [28, 29].

A possible explanation for the link between problem gambling and other addictive behaviours are underlying psychological mechanisms including the concept of a potential ‘reward deficiency syndrome’ [66]. Proposed by Blum and colleagues, this concept links all addictive, compulsive and impulsive behaviours by postulating that a specific genetic variant that is responsible for creating dopamine receptors in the brain can result in a heightened risk for addiction. Furthermore, if at risk for one addition, it is hypothesized that the individual is also at a higher risk for other addictions, which explains why those with problem gambling behaviour have a higher likelihood of developing other addictive, compulsive or impulsive, and dysfunctional behaviours [66]. Similar to the previously discussed disorders, impulsivity has been shown to play a role in behavioural addictions, characterizing both problem gamblers and gamers [25]. The evidence found in the present study of the association, between problem gambling and each of the other behavioural addictions assessed, lends support to the addiction syndrome model, which views addiction as a syndrome that shares common etiological vulnerabilities. The model asserts that there is a singular addiction with multiple expressions and proposes a more comprehensive philosophy of addiction [67]. The validation of such a model has the potential to “increase efficiency in treatment delivery by targeting common etiological variables across multiple disorders with a single, unified treatment” [19]. Due to the paucity of studies on the association between problem gambling and other addictive behaviours, future research should strive to gain a deeper and more complex understanding of the common underlying etiology between these co-morbid addictive behaviours.

### Implications

The present study may have several implications for the health care system and for future research in the area. The demonstration of the heightened level of health issues possessed by problem gamblers reinforces the need for integrative screening and treatment in other relevant healthcare settings. The intervention opportunity presented by problem gamblers seeking treatment for comorbid conditions in primary care confirms the importance of surveillance and screening for gambling symptoms in these settings. Additionally, the association between screening positive for lifetime problem gambling and a range of addictive behaviours supports the belief that settings working with mental health issues may need to improve their awareness of and attention paid to problem gambling in the context of other addictive patterns and mental health. Furthermore, settings in which gambling disorder is treated should also ensure screening for psychiatric diseases; although associations with the psychological distress screening in the present study demonstrated independent associations only for the moderate degree of distress, the unadjusted difference in prevalence of psychological distress was large, when comparing lifetime problem gamblers to non-problem gamblers in the study.

Equally important to identifying cases of problem gambling in diverse healthcare settings, is creating integrative treatment strategies that address the unique complications of individuals diagnosed with comorbidities in addition to problem gambling. The evidence provided by the present study motivates the necessity for effective treatment that addresses multiple diagnoses and implements an integrated and collaborative approach in its execution. For example, the clear association with tobacco smoking may enhance the need to better address health hazards related to smoking in problem gambling patients.

In future research, more longitudinal and prospective studies must be conducted to provide an improved knowledge of the temporality of problem gambling and its comorbidities to gain an understanding of the underlying mechanisms behind the etiology of these co-occurring conditions. To improve the clinical utility of gambling studies, more research on the pathways of development and course of illness should be conducted, as it is imperative in the improvement of current and future treatment delivery. As an extension of understanding common etiological pathways for co-morbidity in disordered gambling, an examination of the bi-directional effects of comorbidity should also be a priority in future research. Likewise, the pathways model may be useful both in a clinical setting and in future longitudinal research studies, in order to better highlight the etiology of problem gambling in an individual, for example with respect to psychological and socio-demographic risk factors. Here, this may include the use of a structured screening instruments, such as the Gambling Pathways Questionnaire [68].

### Strengths and limitations

The key strengths of the present study include the nature of the study being conducted in a broad sample aimed to include people derived from the general population, rather than a clinical setting of treatment seeking problem gamblers. The applicability of results to other populations, however, should be considered with caution. Another strength is the assessment of a wide range of mental health disorders and other co-occurring conditions, as the authors were able to collect data on substance use, mental health, and non-substance-related addictive behaviours, in addition to a spectrum of gambling behaviour. Utilizing a general health survey with multiple health conditions allows the issue of problem gambling to be analyzed in a broader health perspective.

The use of an online survey could be considered both to be a strength and a limitation for various reasons. The use of self-administered questionnaires for sensitive topics, such as mental health, substance use and other addictions, are superior to face-to-face interviews as the perceived impersonality may encourage reporting of sensitive information and lead to higher levels of reporting [69, 70]. Furthermore, self-administered questionnaires can be advantageous for the avoidance of social desirability bias, as possible social cues demonstrated by the researcher that could influence the respondent are removed, therefore also negating the possible need for approval [69]. Conducting the survey in an online format also possesses limitations in terms of sampling due to potential selection bias, which may be a contributing factor towards the higher prevalence of problem gambling reported in this sample than the general population. It can be discussed whether an online survey would bias the prevalence of problem gambling found, compared to face-to-face or postal data collection methods. Previous online surveys in the present setting have tended to report relatively high rates of lifetime problem gambling [71, 72] compared to the general population [73]. This leads to the suspicion that an online survey in this area could potentially attract people with more intensive gambling habits. In contrast, in the present study, the prevalence of problem gambling– 5.7 percent screening positive for a lifetime history–was lower than in previous studies, and may therefore be closer to the general population. Despite this, a selection bias from conducting a survey online and from an online panel cannot be excluded. Although a representative sample was selected, the use of online surveys systematically excludes candidate population members who are not internet users, and additionally, there may be systematic differences between the general population and the respondents who had signed up to receive regular web surveys, as this panel may possess different internet habits. The potential relation between increased gambling habits and being prone to engage in web surveys, as evidenced by the link between problematic internet use and increased problem gambling [74], could represent a problematic relation in the present study. Additionally, there is evidence of a positive correlation between gambling behaviour and the decision to participate in gambling surveys, as web surveys addressing this area of research may attract those with an increased interest in gambling [75]. Although these limitations remain important considerations, studies conducted on the validity of web-based surveys through the comparison of their results to those of identical studies conducted in the real world have found that “the validity and reliability of data obtained online are comparable to those obtained by classical methods” [76].

The validity of self-reported physical activity and BMI is also a limitation of the present study, as participants may have an inaccurate perception of these factors due to denial or exaggeration. However, the accuracy of self-reported health and levels of exercise is supported by previous research [77, 78]. Another significant limitation of the present study is the inability to establish temporality due to the concurrent assessment of gambling addiction and the co-occurring health conditions, therefore preventing determination of the causal pathway. The paucity of information regarding the temporality of these conditions is an issue that has been addressed in the existing literature, as “the mechanisms underlying co-morbidity in disordered gambling and common etiological pathways” are not currently understood [19]. Therefore, it would be beneficial for future research to address the issue of disorder onset.

Although the survey was able to address a spectrum of co-occurring disorders and conditions, due to length constraints put in place to maximize response rate, diagnostic scales utilized to test for the variables of interest were mostly abbreviated versions of the original, consisting of fewer questions. It may be hypothesized that using the full versions may improve sensitivity and specificity, however research has shown that the modified versions perform similarly in comparison to the full versions and are useful as brief assessment tools for their relevant conditions/disorders. Brief screening tools are important in general population health surveys when several topics are being explored, due to the time constraint of how long participants are willing to spend on completing a survey.

As the present study aimed for a brief screen of problem gambling and other addictive conditions including behavioural addiction, brief screening scales were primarily included rather than actual diagnostic scales. This is potentially a limitation, and conclusions of the study should be seen in light of this, and related to this, it should be borne in mind that the time perspective of the chosen screening scales are different. Thus, conclusions drawn from the present analyses specifically apply to recent health variables associated with a lifetime history of problem gambling.

## Conclusions

The present study has confirmed the association between screening positive for a history of problem gambling, and a variety of other behavioural addiction, such as problematic shopping and problem gaming, as well as with moderate psychological distress and tobacco smoking, while controlling for a number of socio-demographic and health-related variables. Likewise, screening positive for lifetime problem gambling was significantly more common in men and respondents with lower level of education. The confirmation of such association bolsters the concept of gambling disorder as a relevant public health issue that should be viewed in the context of public health, requiring the attention of health practitioners, policy makers, and public health strategists. The conclusions derived from the present study have implications for the healthcare system and the treatment of problem gambling and its comorbid disorders, and enhance the need to screen for and address problem gambling in the setting of other addictive behaviours and health.
